# Supplementary material for: A Fine Regulation of the Hippocampal Thyroid Signalling Protects Hypothyroid Mice against Glial Cell Activation
Source: Int J Mol Sci. 2022 Oct 8;23(19):11938. doi: 10.3390/ijms231911938 (PMC9569489; doi:10.3390/ijms231911938)
Supplement: Supplementary file 1 [file ijms-23-11938-s001.zip › ijms-1895532-supplementary.pdf]

Supplementary Materials

Table S1. Summary table of the statistics (p-value) for TH signaling (related to Figure 1), glial cells (related to Figure 2-3) and Open Field test (Figure 5). Statistical non-parametric two-way ANOVA with permutations tests (strain; treatment; Strain*treatment effects) were performed followed by post-hoc analysis (indicated on each graph). IHC: immunohistochemistry. ns: not significant.

	Parameter	Strain	Treatment	Strain*Treatment
			p-value	
**THs level (Figure 1)**	**T4**	**ns**	0.00019	< 0.00001
**TH signaling genes (Figure 2)**	*Trα1*	0.00019	ns	0.001
	Trβ1	ns	ns	0.006
	Dio2	ns	ns	0.001
	Dio3	ns	ns	0.001
	Rc3	ns	0.017	0.014
	Klf9	0.00039	ns	< 0.00001
**Reactive astrocytes and activated microglia (western blot analysis; Figure 3-4)**	STAT3	0.0075	ns	ns
	P-STAT3	ns	ns	0.006
	Ratio P-STAT3/STAT3	ns	ns	0.003
	CD11B	0.0061	ns	< 0.00001
	IBA1	ns	ns	ns
**Glial cells markers (IHC analysis; Figure 3-4)**	IBA1	0.0001	ns	0.041
	CD68	ns	0.00019	< 0.00001
	GFAP	0.00019	ns	< 0.00001
**Open Field test (Figure 5)**	Distance in center	ns	ns	0.035
	Time in center	ns	ns	0.015
	Velocity in center	0.0039	ns	0.025

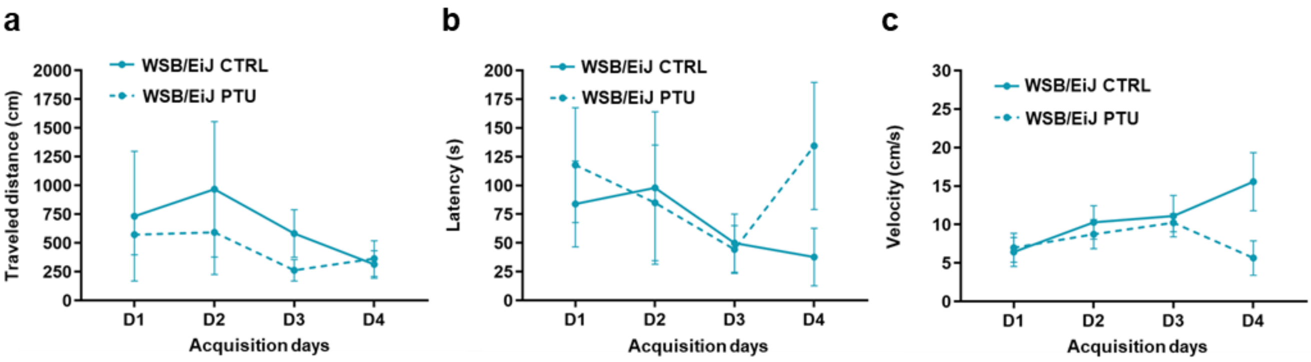

Figure S1. Learning assessment in WSB/EiJ mice after PTU-treatment. Measurement of the traveled distance **(a)** latency **(b)** and velocity **(c)** to escape into the box of the Barnes maze during the four acquisition days, evaluating learning performances of euthyroid (CTRL) and hypothyroid (PTU) WSB/EiJ mice (n = 3-4 per group). No statistical analysis was performed because Barnes maze test was not completed on this strain. The data are expressed as mean ± SEM.
